# Research progress on plant-derived bioactive compounds and late-life depression: from neuroprotective mechanisms to dietary patterns

**DOI:** 10.3389/fnut.2026.1913204

**Published:** 2026-09-02

**Authors:** Yao Gao, Yu-Fei Shi, Jian-Zhen Hu, Ze-Kun Li, Xin-Zhe Du, Sha Liu

**Affiliations:** 1 Department of Psychiatry, First Hospital/First Clinical Medical College of Shanxi Medical University, Taiyuan, China; 2 Shanxi Key Laboratory of Artificial Intelligence Assisted Diagnosis and Treatment for Mental Disorders, First Hospital of Shanxi Medical University, Taiyuan, China; 3 Department of Mental Health, Shanxi Medical University, Taiyuan, China; 4 Nursing College, Shanxi Medical University, Taiyuan, China

**Keywords:** late-life depression, neuroprotection, plant-derived bioactive compounds, polyphenolic components, terpenoid components, dietary patterns

## Abstract

Late-life depression (LLD) represents a critical public health challenge, exerting a profound and detrimental impact on the health of older adults. Currently, dietary intervention is increasingly valued in the field of mental health. Various active components abundant in plant-based foods show great potential in preventing and alleviating depressive symptoms in the older adults. This narrative review aims to summarize the neuroprotective effects of major plant-derived bioactive compounds such as polyphenols, terpenoids and other components, which are related to LLD. It also listed alkaloids and polysaccharides as potential plant-derived bioactive compounds for LLD. It focuses on elucidating the research progress of their mechanisms and dietary patterns, thereby providing a theoretical basis for dietary intervention in treating older adults who are over 60 years old. Future trials should integrate multi-omics and quantitative evaluation to inform personalized LLD dietary interventions.

## 1 Introduction

Late-life depression (LLD) is a common affective disorder with high prevalence and high disability rates (1). Patients with depression over 60 years old are often referred to LLD (2). It not only severely impairs the quality of life and functional independence of older adults, but is also often comorbid with cognitive decline and various physical diseases, posing complex clinical challenges (3). Studies show that LLD patients often have cognitive impairment, which may persist even after depressive symptoms remit, increasing the risk of progression to dementia (4, 5). Traditional antidepressant drugs have limitations such as slow onset, multiple side effects, and high relapse rates (6, 7). Therefore, exploring safe, effective, and accessible preventive and adjunctive potential intervention strategies is crucial for improving the prognosis of LLD.

In recent years, the rise of nutritional psychiatry has emphasized the key role of dietary patterns in intervening in psychiatric disorders (8, 9). plant-derived bioactive compounds and plant-based dietaries regulate neurobiological pathways closely related to depression through multi-target effects, possessing anti-inflammatory and antioxidant properties (10). Diets rich in fruits, vegetables, and whole grains may offer neuroprotective effects at a macroscopic level. Investigating plant-derived bioactive compounds as intervention strategies has both theoretical and practical significance, and it provides fresh perspectives for treating LLD.

**FIGURE 1:**
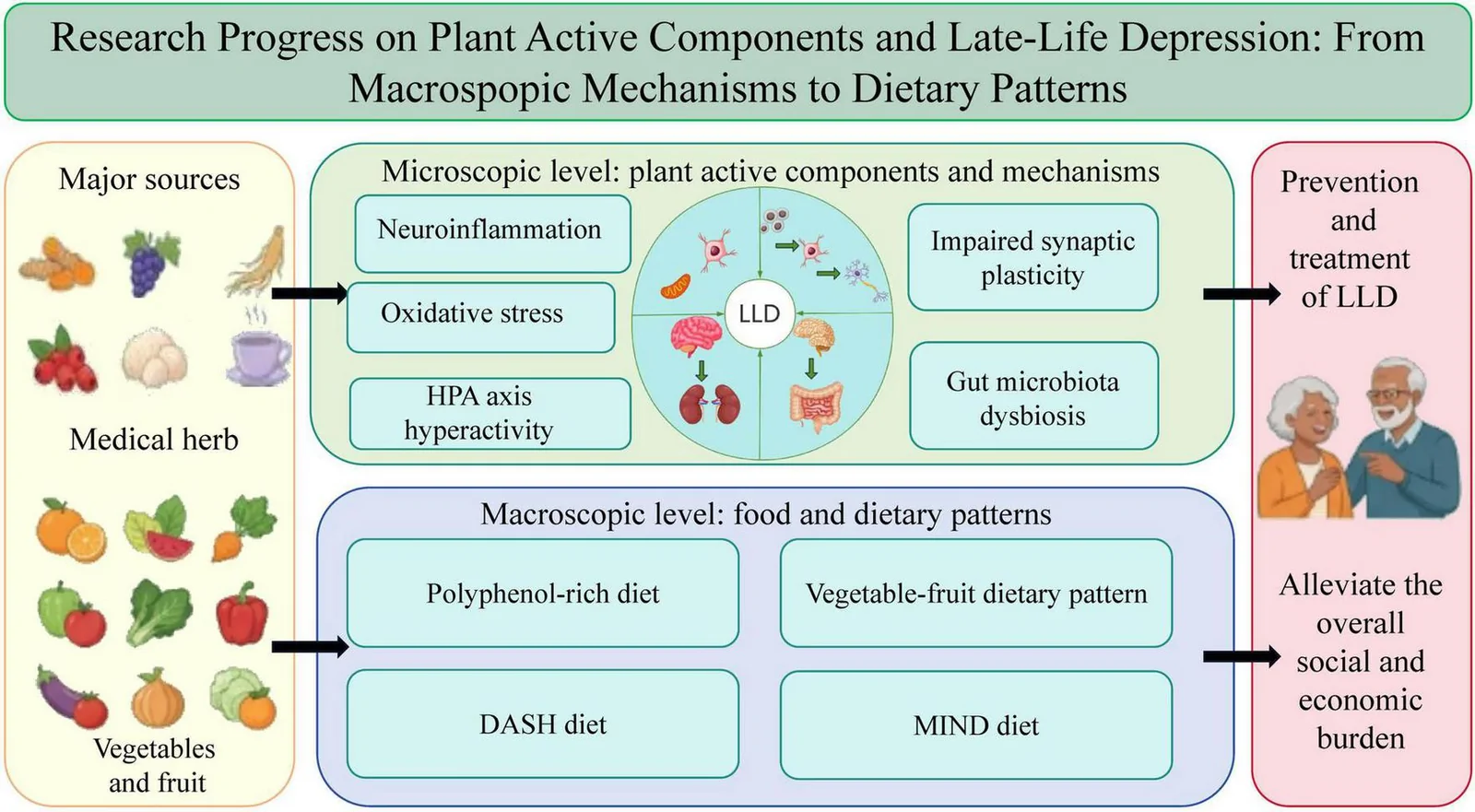
Mechanisms and dietary patterns of major plant-derived bioactive compounds potentially related to LLD. DASH diet, Dietary Approaches to Stop Hypertension diet; MIND diet, Mediterranean-DASH Intervention for Neurodegenerative Delay diet.

However, current reviews predominantly examine antidepressant effects in general depression, overlooking the specific context of LLD (11–13). Therefore, there remains a research gap regarding the efficacy of plant-derived bioactive compounds for the treatment of LLD. This article systematically elaborates the research progress from microscopic molecular mechanisms to macroscopic dietary patterns, with the objective of providing a scientific basis for reducing the risk of depression and potential intervention for LLD based on dietary intervention (Figure 1).

To ensure the quality of our review, we rigorously select the source. Since PubMed is a comprehensive biomedical database and China National Knowledge Infrastructure (CNKI) is the most widely used database in China, which contains plant active ingredients from traditional Chinese medicine, we chose PubMed and CNKI databases for literature search, retaining the literature directly related to LLD, as well as some related to common depression. Extrapolate the relevant literature on neurodegenerative diseases, etc., We also used levels of evidence from the Oxford Centre for Evidence based Medicine^1^ to conduct a quality assessment (14).

## 2 Major plant-derived bioactive compounds for potentially modulating mechanisms related to LLD

This section provides an overview of the major plant-derived bioactive components that have the potential to modulate key mechanisms implicated in LLD: There are many types of plant-derived bioactive compounds, mainly classified by chemical structure into polyphenols, terpenoids, and other active components, which exert antidepressant effects through different mechanisms. Representative components and their main findings are detailed in Table 1. Table 1 only about the plant-derived bioactive compounds directly related to LLD, due to the temporary lack of evidence that alkaloids and polysaccharides are directly related to LLD, we have not listed these two plant-derived bioactive compounds in it. For fungi (such as Ganoderma lucidum and Hericium erinaceus) and algae (astaxanthin), although they do not belong to plants, we retain them as potential components because their mechanism of action is related to the pathological mechanism of LLD.

**TABLE 1:** Summary of key studies on plant-derived bioactive compounds and late-life depression.

Plant-derived bioactive compounds	Major sources	Main findings	Evidence level	References
Polyphenols	Flavonoids, blueberries, anthocyanins,	Flavonoid intakes lower depression risk	Moderate (prospective cohort)	(20, 47, 68)
Tea polyphenols	Green tea	GTP might improve LLD	Low (animal experiment)	(51)
Terpenoids	Ginseng	Ginseng improves cognitive function for LLD	Moderate (prospective cohort)	(26)
Volatile oils	Lavender, broccoli	Lavender tea reduces depression scores	High (RCT)	(42)
Ginkgo biloba extract (EGb761)	*Ginkgo biloba L. leaf*	EGb 761 has the potential to manage LLD	Low (animal experiment)	(39)
Lutein	Spinach, parsley, chard, broccoli, zucchini, and peas	Higher lutein intakes improve depressive symptoms	Moderate (prospective cohort)	(40)
Kaempferol	Kale, endive, leeks, turnip greens, and tea	Kaempferol improves depressive symptoms for aged rats	Low (animal experiment)	(41)

### 2.1 Polyphenolic components and LLD

Polyphenolic components, derived from flavonoids, curcuminoids, blueberries and compound preparations, are credited with antidepressant actions driven by their antioxidant and anti-inflammatory mechanisms (15). The representative components of polyphenols also include tea polyphenol, green tea polyphenol (GTP) and anthocyanins. Among numerous polyphenols, quercetin, epicatechin, resveratrol, and curcumin have been recognized as the compounds exhibiting the greatest neuroprotective activity (16). Curcumin, in particular, has been widely used in treating depression and cognitive impairment (17, 18). A cohort study on aging shows that consuming foods rich in curcumin can lower the Geriatric Depression Scale (GDS) score and improve depressive symptoms (19). Flavonoids also play an essential role in improving the depression symptoms, especially for old women (20). And anthocyanins, a plant active component belongs to flavonoids, can delay cognitive aging (21). An research indicates that polyphenol-rich dietary patterns are significantly associated with older adults with depression but it is a indirect evidence (22). A 12-week placebo-controlled trial has shown that dietary supplementation with isoflavones and resveratrol may improve depressive symptoms in women aged 50 to 55 (23). Therefore, incorporating polyphenolic compounds into the diet or using them as adjunctive therapy provides a safe and multi-beneficial new strategy for preventing and managing LLD.

### 2.2 Terpenoid components and LLD

Terpenoid components, mainly derived from ginseng and Ganoderma lucidum, are increasingly gaining attention in regulating the hypothalamic-pituitary-adrenal (HPA) axis, with their structural diversity and broad biological activity (24). Triterpenoids, represented by ginsenosides from ginseng and ganoderic acids from Ganoderma lucidum, have been shown to stabilize HPA axis function through multi-level interventions. They can act on the hypothalamus to regulate corticotropin-releasing hormone (CRH) secretion or enhance the feedback sensitivity of hippocampal glucocorticoid receptors, thereby inhibiting excessive HPA axis activation and reducing circulating stress hormone levels (25). A study proved that a cumulative duration of ginseng intake greater than 5 years might represent a viable strategy for cognitive maintenance in late life by investigating community-dwelling elders in Korea but it is still an indirect cognition-related evidence (26). It is increasingly evident from existing data that terpenoids have a role in mood modulation., alleviating anxiety and improving cognitive function in LLD, having a potential to lay a solid foundation on developing novel antidepressant therapies based on plant-derived bioactive compounds (24, 27).

### 2.3 Alkaloid components and LLD

The main sources of alkaloids are *Coptis chinensis*, *Phellodendron amurense*, and their compound formulas. Their mechanism of action is similar to traditional drugs but with higher tolerability (28). Among these, berberine has been particularly extensively studied. Berberine derived from Coptis chinensis. It can easily traverse the blood-brain barrier and exhibits neuroprotective potential in various neurodegenerative and neuropsychiatric disorders, including depression and anxiety (29). Although limited research has directly examined the link between alkaloids and LLD, LLD exhibits a high comorbidity with neurodegenerative disorders (30–32) and alkaloids such as berberine can improve the symptoms of neurodegenerative disorders. It may be a potential intervention for LLD. Another representative component is betaine. And it is worth noting that betaine may be a biomarker in depression Fecal Microbiota Transplantation (FMT) rats (33, 34). Future studies are warranted to examine the efficacy of alkaloids in geriatric depression and to clarify the mechanistic pathways involved.

### 2.4 Polysaccharide components and LLD

Polysaccharide components, mainly derived from *Hericium erinaceus*, *Lentinus edodes*, and *Lycium barbarum*, also show potential in intervening in LLD. Given their antioxidative, neuroinflammation-resolving, and neuroprotective capacities, extracts from Hericium erinaceus have effects attributed to depression (35). *Lentinan* and *Lycium barbarum* polysaccharides also fall into this category. They may influence brain function by regulating the “gut-brain axis” (36). What is more notable is that disaccharides such as sucrose may instead induce depressive symptoms in the older adults. A study involving elderly people in the community (with an average age of 75) indicated that depressive symptoms were associated with a higher intake of sucrose (37). Although there are not enough studies about the direct evidence on polysaccharide and LLD, here are emerging evidence identifying the gut-brain axis and gut microbiota as a promising therapeutic for LLD (38), which suggests that polysaccharides might indirectly modulate central nervous system inflammation and stress responses by improving the gut microecology, offering a new perspective for dietary intervention in LLD.

### 2.5 Other plant-derived bioactive compounds and LLD

Other types of plant-derived bioactive compounds such as volatile oils, Ginkgo biloba extract (EGb761) (39), Lutein (40) and kaempferol (41) also deserve attention. For example, the main active components of lavender include volatile oils, luteolin, etc., Lavender herbal tea consumption appears to be effective in lowering depressive and anxiety scale ratings (42), and its aromatic oil has been proven useful in geriatric depression care (43). Lavender is inexpensive and easily accessible, serving as an adjunctive treatment to alleviate anxiety and depression in older adults.

## 3 Research on the mechanisms of major plant-derived bioactive compounds potentially related to LLD

### 3.1 Neuroinflammation

Neuroinflammation has emerged as a prominent pathophysiological determinant in the etiology of LLD (44). Inflammatory indicators are reported to play a mediating role in the connection between anti-inflammatory diets and depressive disorders. Adopting an anti-inflammatory diet may help lower the likelihood of depression in middle-aged and older adults (45). Plant-derived bioactive compounds, such as astaxanthin, curcumin, quercetin, and resveratrol, achieve neuroprotection and delay the process of aging (46) by targeting and regulating neuroinflammatory signaling pathways. For example, the blueberry is the source of anthocyanins and flavonoids. A protocol of randomized, placebo-controlled pilot study found that blueberries may improve depressive symptoms in old people by reducing motivation loss caused by neuroinflammation (47). Another representative component, curcumin, an extensively studied plant polyphenol, acts by directly interacting with the IKK (Inhibitor of nuclear factor kappa-B kinase) complex, thereby inhibiting the phosphorylation and degradation of Inhibitor of κappa B (IκB), blocking the nuclear translocation and activation of Nuclear Factor kappa-B (NF-κB), and ultimately reducing the production of downstream pro-inflammatory mediators (17). Although it can improve cognitive impairment in older adults, whether it is effective for LLD needs further exploration. These precise molecular interventions effectively mitigate the disruption of blood-brain barrier integrity caused by neuroinflammation.

### 3.2 Oxidative stress

The aging brain is often accompanied by elevated levels of oxidative stress, leading to neuronal damage and dysfunction. Various plant active ingredients, such as herbal terpenoids, thymol and carvacrol, delay aging by protecting mitochondria from oxidative stress (48). Plant-derived bioactive compounds can effectively enhance the body’s endogenous antioxidant defense pathways, exerting neuroprotective effects (49). A study showed that Ginkgo biloba extract (EGb761) produced antidepressant-like actions by mitigating neurotransmitter disruptions and reestablishing redox balance in the cerebral cortex, demonstrating potential for managing LLD (39). Researches on plant-derived bioactive compounds which treat LLD by the role of reducing Oxidative stress could go back to about 14 years ago. A cross-sectional dietary survey revealed that older adults with higher dietary lutein intake were less likely to report clinically significant depressive symptoms, even after adjusting for potential confounders (40). These basic studies provide important theoretical bases for developing neuroprotective strategies based on natural products.

### 3.3 HPA axis hyperactivity

Patients with LLD often exhibit chronic overactivation of the HPA axis, leading to persistently elevated circulating cortisol levels, which in turn damages hippocampal neurons and weakens their negative feedback inhibition on the HPA axis, forming a vicious cycle (50). A study in 2011 found that green tea polyphenols (GTP) might improve LLD by inhibiting HPA (51). Many other plant-derived bioactive compounds can directly act on key nodes of the HPA axis. For example, ginsenoside Rg1 can inhibit the excessive internalization of the glucocorticoid receptor (GR), restoring the sensitivity of hippocampal GR to cortisol (52). Ginsenoside Rg3 could regulate both HPA and sympathetic-adrenomedullary system (SAM) (53). Aging process is related to HPA, which means it may be a specific mechanism comparing to teenagers and adults (54). By multi-target intervention of HPA axis hyperactivity, plant-derived active molecules have the potential to block the “stress-cortisol-neural damage” pathological chain, offering safer treatment options for elderly depression patients.

### 3.4 Impaired synaptic plasticity

Aging plays a negative effect on the decline of neural plasticity. Older adults with depression have the characteristics of neurogenic damage and abnormal functional connectivity (55, 56). Existing evidence has shown that long-term intake of foods or extracts rich in specific plant-derived bioactive compounds can effectively enhance the proliferation and differentiation of neural precursor cells in the hippocampal dentate gyrus, which counteracts stress- or aging-induced suppression of neurogenesis. For instance, tea polyphenol (TP) mitigated depression-like behaviors in elderly rats via modulation of BDNF expression (57). A Chinese study proved that kaempferol can also improve depressive symptoms in elderly rats with chronic stress-induced depression models by increasing the expression of BDNF (41). This evidence clearly indicates that plant-derived bioactive compounds can strengthen synaptic connections structurally and improve synaptic transmission efficacy functionally, which is a core mechanism underlying their neuroprotective and antidepressant effects for LLD.

### 3.5 Gut microbiota dysbiosis

Normal brain aging is typically associated with cognitive decline, which is largely driven by the aforementioned mechanisms, and these pathological processes are further aggravated by gut microbiota dysbiosis (58). The gut microbiota shows a significant change during aging and age-related diseases with its composition and function, which is a novel therapy for LLD (59–61). And an experiment enrolling 644 participants additionally supported the gut microbiota to be a bridge between plant active component-based dietary and LLD (62). Selective promotion of beneficial gut microbiota growth is achievable through diets characterized by high polyphenol content (41, 63). Plant-derived bioactive compounds such as plant-derived extracellular vesicles (EVs) is a novel strategy for depression by targeting the gut-brain axis (64). Liu found that betaine may improve depression through the gut-brain axis (65). However, evidence on it is still warranted.

The molecular mechanisms of the above-mentioned single components provide biological rationality for explaining the protective effect of macroscopic dietary patterns (Table 2). However, it is difficult for a single component to reach the clinical therapeutic concentration. The multi-component synergy effect in Whole Foods and the improvement of bioavailability of food matrices may be the key for dietary patterns to exert long-term benefits.

**TABLE 2:** Pathological mechanisms of LLD and targeted effects of plant-derived bioactive compounds.

Pathological mechanism	Key molecules/conclusion	Representative phytoactive components	Major sources	Dosage	Reported outcomes	References
Neuroinflammation	Motivation loss caused by neuroinflammation	Anthocyanins, flavonoids	Berries (44)	48 g of freeze-dried blueberry powder (600 mg of anthocyanins and 8 g of fiber)	Improve depressive symptoms in old people	(47)
Oxidative stress	ROS	EGb761, EGCG	Ginkgo biloba leaf (47)	High-dose EGb761 (200 mg/kg/day)	Activates endogenous antioxidant enzymes, scavenges free radicals	(39)
	Antioxidant	Lutein	Spinach, parsley, chard, broccoli, zucchini, and peas (103)	1681.9 μg/day	Improves depressive symptoms	(40)
HPA axis hyperactivity	Inhibition of HPA axis activity	GTP	Green tea (leaves or brewed beverage) (104)	5, 10 and 20 mg/kg	Suppresses excessive stress response, restores negative feedback	(51)
Impaired synaptic plasticity	The expression of BDNF	Tea polyphenol, kaempferol	Tea polyphenols: green/black tea;kaempferol: kale, endive, leeks, turnip greens, and tea (38, 59)	300 mg/kg, 50 mg/kg	Promotes neurogenesis, increases dendritic spine density	(41, 57)
Gut microbiota dysbiosis	The regulation of metabolites such as 3-hydroxybenzoic acid and the gut-brain axis	Polyphenols	Grape seed	15 g/L	Reduce anxiety-like behaviors induced by oxidative stress and improve cognitive function	(63)
	Gut-brain axis	Betaine	Wheat bran/germ, spinach, whole-wheat bread, and other wheat foods (105)	Animal: 600 mg/kg/day, people: 400 mg/day	Improves idepression and anxiety	(34)

ROS, Reactive Oxygen Species; EGb761, *Ginkgo biloba L*. leaf extract; EGCG, Epigallocatechin Gallate; GTP, green tea polyphenols; BDNF, Brain-Derived Neurotrophic Factor. So far, no plant-derived bioactive compounds have been found to treat LLD by regulating the gut microbiota and the gut-brain axis. Both polyphenols and betaine targeting gut-brain axis in this table are related to common depression.

## 4 Plant active component-based dietary interventions and late-life depression

Building upon these preliminary findings, we broaden our unit of analysis from isolated phytochemicals to holistic dietary patterns. A longitudinal study of adults aged 55 and over found that healthier dietary patterns were associated with lowering the risk of depressive symptoms (66). Our review focuses on epidemiological evidence for plant-based dietary patterns, with particular attention to disaggregating the heterogeneity between healthy and unhealthy variants of such diets (Table 3).

**TABLE 3:** Four major dietary patterns of plant-derived bioactive compounds to LLD.

Dietary patterns	Major sources of plant-derived bioactive compounds	Core mechanisms	Participants	Clinical tools	Level of evidence	Reference
Polyphenol-rich dietary	Flavonoids and blueberries	Anti-inflammatory, gut microbiota remodeling	Older adults	Center for Epidemiologic Studies Depression Scale (CES-D)	Moderate (prospective cohort)	(68)
Vegetable-fruit dietary	Tubers, fruits, bacteria and algae, legumes, vegetables, and nuts	Anti-inflammatory and antioxidant	Older adults	Self-rating Depression Scale (SDS)	Moderate (prospective cohort)	(72)
Dietary Approaches to Stop Hypertension (DASH) diet	Legumes, fruits, and vegetables	Anti-inflammatory and antioxidant	Older adults	The Beck Anxiety Inventory and the Beck Depression Inventory	Moderate (prospective cohort)	(78)
Mediterranean-DASH Intervention for Neurodegenerative Delay (MIND) diet	Fruit, legumes, vegetables, and nuts	Anti-inflammatory and antioxidant	Middle-aged and older individuals	Validated Food Frequency Questionnaire (FFQ)	Moderate (prospective cohort)	(80)

### 4.1 Polyphenol-rich diet: delaying aging and alleviating cognitive impairment

Polyphenol-rich dietary patterns provide various natural plant secondary metabolites, demonstrating unique anti-inflammatory and anti-aging value in the health management of the older adults, along with a preventive effect against neurodegenerative diseases (67). A study supported that high intake of polyphenol related tightly to lower depressive risks in LLD (68). Furthermore, the intake pattern mainly derived from tea and rich in monomer flavanols and theaflavins is associated with a reduced risk of depression in the older adults (68). Although existing research reveals individual differences in polyphenol bioavailability, multi-step interventions such as synergy with dietary fiber, adjunctive probiotic metabolism, and gut microbiota remodeling can effectively enhance their bioactivity and translate into clinical benefits (69). Incorporating polyphenols into daily dietary habits is a promising approach to enhance immune resilience and may reduce chronic diseases.

### 4.2 Vegetable-fruit dietary pattern: preventing age-related disorders

The vegetable-fruit pattern, rich in root vegetables, fruits, fungi and algae, pulses, greens, and nuts, plays an important role in alleviating late-life depressive symptoms (70, 71). A cross-sectional study from rural Tianjin, China, showed that depressive symptoms significantly decreased, with the quartile of the vegetable-fruit pattern increasing (72). A Cross-Sectional Analysis from society additionally proved that intake of vegetables and nuts could lower risks of depression (73). Furthermore, previous researches confirmed that high consumption of vegetables and fruits in one’s diet contributes significantly to lowering the risk of depression (74, 75). Moreover, a study in Singapore supported that individuals may lay a solid foundation that reduces the eventual risk of depressive symptoms in old age by ensuring sufficient fruit intake early on (76). A vegetable-fruit-based dietary structure not only provides an effective and sustainable strategy for dietary intervention in LLD but also prevents age-related visual impairment and dual sensory impairment (77).

### 4.3 Other plant-based dietary patterns: lowering the risk of depressive symptoms

Other dietary patterns rich in plant-derived bioactive compounds-based foods, such as the traditional Nordic diet and the Dietary Approaches to Stop Hypertension (DASH) diet, have also been proved to be a potential diet for emotional health (78). A systematic review integrating 33935 participants further confirmed that people who had DASH dietary were less likely to become depressed (79). In addition, a cohort study showed that adherence to the Mediterranean-DASH Intervention for Neurodegenerative Delay (MIND) diet was negatively associated with the rate of brain structural atrophy, supporting brain health and suggesting its potential for potentially modulating mechanisms related to LLD (80). A nationwide study from China found that the Chinese version of MIND (cMIND) diet can significantly reduce the likelihood of developing depressive and anxiety symptoms for older adults (81). It is worth mentioning that when implementing dietary interventions for older adults, careful consideration must be given to their loss of chewing and swallowing ability (82) and reduced nutrient absorption status (83), for individualized adjustments. Providing appropriately processed food forms (chopped, cooked, pureed) ensures nutrient intake and safety, thereby maximizing the protective outcomes of plant-based dietary patterns in ameliorating LLD.

### 4.4 Unhealthy plant-based diet: increasing the likelihood of depressive manifestations

It is recognized that not all plant-based eating patterns are advantageous for the psychological well-being of older adults; for instance, those high in refined grains, sugary drinks, sweets, and fried potatoes may be less favorable. Although these foods are technically derived from plants, they lack dietary fiber and bioactive components, and are often high in added sugar, salt, or unhealthy fats, which is known for unhealthy Plant-Based Diet Index (uPDI) (84). Research has shown that an elevated uPDI is linked to an increased likelihood of depressive manifestations in older adults (85). Another longitudinal study across from 10 years in community-dwelling older adults also showed that those in the highest uPDI category were more inclined to show a depressive symptom trajectory (86). Moreover, a Chinese Longitudinal Healthy Longevity Survey derived from 11,437 older adults proved that flexitarian dietary patterns increased the risk of depressive symptoms (87). This evidence suggests that simply emphasizing “plant-based” while ignoring the degree of food processing and nutritional composition may lead to misleading conclusions.

### 4.5 Bioavailability and polypharmacy

In clinical nutrition, bioavailability and polypharmacy play an important role in older adults. Polyphenol has strongest LLD evidence, but it has low bioavailability. Although curcumin, resveratrol and other components have significant neuroprotective effects, due to their poor water solubility, strong first-pass effect and limited blood-brain barrier penetration ability, their blood drug concentration and brain tissue distribution are insufficient (88). More importantly, the interactions between different components and their specific effects are still unclear (89). St. John’s wort (Hypericum perforatum), as a medicinal plant with an application history of 2,000 years, has better tolerance than tricyclic antidepressants and has the potential to become a substitute for benzodiazepines (90). However, a previous study has shown that taking St. John’s wort simultaneously with p-glycoprotein (P-gp) substrate drugs targeting the central nervous system (CNS) does not lead to P-GP-mediated drug interactions at the blood-brain barrier, resulting in a reduction in the exposure and efficacy of certain P-gp substrate drugs targeting the CNS in the brain (91). In clinical nutrition, the interaction between drugs and food is particularly important. Older adults with depression often take antidepressant drugs (92). Exploring the interaction between drugs and plant active ingredients is an important safety consideration.

Dietary pattern is a major determinant of health (93). For the older adults, they offer a more convenient way to adjust and optimize health. The above cohort studies indicate that consuming curcumin-rich curries once a month or once a year has the most significant effect in reducing GDS scores (19) and consuming at least one serving of citrus fruits and fruit juice daily or eating apples at least five times a week can most significantly improve depressive symptoms in the older adults (20). These evidence for the plant-based dietary provides strong support for the “food is medicine” concept. Adhering to reasonable and diverse dietary patterns may be an important and economical way to protect against LLD.

## 5 Current status of clinical research and application challenges

### 5.1 Limitations and challenges of existing research

Current clinical research evidence on plant-derived bioactive compounds for treating LLD has significant limitations. Firstly, most studies have small sample sizes and short durations, leading to inconsistent evidence levels. For example, a meta-analysis on Bacopa monnieri pointed out that the improving trend of this plant extract on cognitive function was not significant and statistically inconsistent (94). Secondly, the heterogeneity of participants’ baseline nutritional status and genetic background easily lead to inconsistencies in research results (88). Then, LLD is often accompanied by other symptoms such as cognitive dysfunction. A RCT showed that black mulberry extract could improve the cognitive function of patients with Alzheimer’s disease (AD), but had no significant effect on improving their depressive symptoms (95). Clinical treatment should select appropriate plant active ingredients based on the specific symptoms of patients to assist in intervention, promoting the individualized development of LLD treatment and clinical nutrition. Finally, clinical studies specifically targeting LLD patients are very scarce (Supplementary Table S1). Although several experiments have confirmed the antidepressant-like behavior and neuroprotective effects of various plant components like *Hericium erinaceus* and *Gastrodia elata* water extract (96, 97), which may lay a solid foundation on clinical researches.

### 5.2 Future research directions and prospects

Clinical studies indicate poor prognosis for LLD (3). Appropriate nutrition (98) and early adoption and maintenance of healthy dietary patterns have profound public health economic value for preventing or delaying the onset of LLD. Network pharmacology that integrates multi-omics data (such as metabolomics, microbiomics, and transcriptomics) provides a powerful tool for a comprehensive understanding of the complete pathway of plant active ingredients from intake to the exertion of neuroprotective effects (99–101). Two animal experiments confirmed the antidepressant activity of the traditional Chinese medicine compound (102) and total glycosides from *Cistanche tubulosa* (99) through multi-omics mechanisms. Future research first need to deeply integrate technologies such as genomics, metabolomics, and microbiomics to identify elderly subgroups that respond well to specific plant active component interventions. Secondly, it should quantitatively evaluate the interaction properties of different plant active component combinations and elucidate their molecular basis, laying the groundwork for developing precise combination therapy strategies based on plant pharmacology. Finally, promoting plant-derived bioactive compounds-based dietary intervention as a public health measure throughout the entire life course and prioritize conducting high-quality Randomized Controlled Trial (RCTs) specifically for LLD aims to alleviate the overall social and economic burden caused by mental health problems in the older adults.

## 6 Conclusion

In summary, polyphenols have the strongest LLD evidence and best dietary patterns. But they have low bioavailability. Terpenoids show moderate HPA-modulating effects. Alkaloids and polysaccharides lack direct LLD evidence. Polysaccharides act via gut-brain axis and lavender has RCT support. The mechanisms of plant-derived bioactive compounds against LLD encompass anti-inflammation, antioxidation, HPA axis stabilization, promotion of synaptic plasticity, along with regulation of the gut–brain axis via microbiota. Epidemiological and clinical evidence for the plant-derived bioactive compounds-based dietary provides strong support for the “food is medicine” concept. However, most current supporting evidence still comes from preclinical and observational studies. Combining multi-omics and quantitative evaluation to achieve personalized application of plant-derived bioactive compounds or their combinations will contribute significant efforts to improving the mental health for older adults.

## Footnotes

1
https://www.cebm.ox.ac.uk/resources/levels-of-evidence/oxford-centre-for-evidence-based-medicine-levels-of-evidence-march-2009
