# Supplementary material for: Research progress on plant-derived bioactive compounds and late-life depression: from neuroprotective mechanisms to dietary patterns
Source: Front Nutr. 2026 Sep 2;13:1913204. doi: 10.3389/fnut.2026.1913204 (PMC13579663; doi:10.3389/fnut.2026.1913204)
Supplement: Supplementary file 1 [file Table_1.docx]

**Table S1 LLD specific evidence and extrapolation evidence**

| **Evidence category** | **Definition** | **Appropriate interpretation** | **Reference** |
| --- | --- | --- | --- |
| **LLD or older adults with depressive symptoms** | older adults with clinically diagnosed depression | Directly relevant, although strength depends on design and sample size | (20, 26, 37, 40, 42, 47, 68, 72, 76, 79, 80, 106) |
| **Indirect human evidence** | General depression, cognitive impairment, dementia, anxiety, or healthy aging populations | Supports plausibility only | (21, 65, 73-75, 77, 95) |
| **Preclinical evidence** | Aged-animal depression models, non-aged depression models, cellular or mechanistic studies | Mechanistic or hypothesis-generating evidence | (33, 34, 39, 41, 51, 57, 63, 64, 91, 96, 97) |
